# Uncovering a novel function of BTLA on tumor-infiltrating CD8+ T cells

**DOI:** 10.1186/2051-1426-1-S1-O1

**Published:** 2013-11-07

**Authors:** Cara Haymaker, Richard Wu, Krit Ritthipichai, Chantale Bernatchez, Marie-Andree Forget, Jie Qing Chen, Hiu Liu, Ena Wang, Francesco Marincola, Patrick Hwu, Laszlo Radvanyi

**Affiliations:** 1 MD Anderson, Houston, TX, USA; 2 National Institutes of Health, Bethesda, MD, USA; 3 Sidra Medical Research Hospital, Doha, Qatar

## 

Manipulation of T-cell co-inhibitory molecules, such as CTLA-4, PD-1, and BTLA has recently moved to the forefront of cancer immunotherapy. Although these molecules serve as inhibitors of T-cell activation, they are also biomarkers for activated T cells and may in fact have positive immune regulatory functions under certain circumstances. Recently, we demonstrated an unexpected positive association of CD8+ T cells expressing BTLA (B- and T- lymphocyte attentuator) with clinical response to adoptive T cell therapy in late-stage melanoma patients. We hypothesized that TIL may utilize the BTLA checkpoint differently and that key phenotypic and functional differences may exist between CD8+BTLA+ and CD8+BTLA- TIL subsets. In this study, we isolated and characterized BTLA+ and BTLA- CD8+ TIL from melanoma patients accrued in a Phase II clinical trial. We found that CD8+BTLA+ TIL had a superior proliferative response to IL-2 and a younger, more central memory T-cell behavior, such as secreting their own IL-2 after TCR stimulation. This younger and more robust memory phenotype was also associated with a longer persistence of T-cell clones in vivo in patients from the infused CD8+BTLA+ TIL subset. In contrast, CD8+BTLA- TIL were poorly proliferative, expressed killer-cell immunoglobulin-like receptors, and exhibited a gene expression signature of T cell deletion. As previously demonstrated, BTLA ligation with its cognate ligand herpes virus entry mediator (HVEM) resulted in decreased proliferation and inflammatory cytokine secretion. However, in a model of TIL activation induced cell death, we observed an enhanced survival of TIL co-cultured with HVEM+ target cells suggesting that BTLA ligation may also promote T-cell survival. Using a recombinant HVEM-Fc protein, we found that HVEM ligation of BTLA on CD8+ TIL activated the PI3K-Akt pathway resulting in the phosphorylation of Akt; this response was blocked using an anti-BTLA antibody. PI3K-Akt activation is most likely mediated by a unique GRB2-binding domain that exists in BTLA that can recruit PI3K via GRB2. As HVEM is expressed by many melanoma tumors and antigen-presenting cells these results suggest that HVEM ligation of BTLA on CD8+BTLA+ effector T-cells in the tumor microenvironment may play a dual role by reducing over-stimulation through the TCR and driving a PI3K-Akt-induced cell survival pathway. This survival signaling pathway may facilitate the longer persistence of the CD8+BTLA+ TIL subset in vivo in our patients. Our study has uncovered a novel role for BTLA both as a biomarker in TIL therapy and as a rheostat in perhaps fine tuning CD8+ T-cell responses rather than simply as a negative signaling pathway.

